# Effects of Small Gaps on the Relationship Among Soil Properties, Topography, and Plant Species in Subtropical Rhododendron Secondary Forest, Southwest China

**DOI:** 10.3390/ijerph16111919

**Published:** 2019-05-30

**Authors:** Fenghua Tang, Wenxuan Quan, Chaochan Li, Xianfei Huang, Xianliang Wu, Qiaoan Yang, Yannan Pan, Tayan Xu, Chenyu Qian, Yunbing Gu

**Affiliations:** 1 Guizhou Provincial Key Laboratory for Information Systems of Mountainous Areas and Protection of Ecological Environment, Guizhou Normal University, Guiyang 550001, China; win_195ss@163.com (F.T.); wenxuanq@gznu.edu.cn (W.Q.); hxfswjs@gznu.edu.cn (X.H.); wuxianliang1995@163.com (X.W.); yangqiaoanyqa@gmail.com (Q.Y.); panyannan528@163.com (Y.P.); xutayan@163.com (T.X.); qianchenyu94@163.com (C.Q.); guyunbing306@163.com (Y.G.); 2 State Key Laboratory of Environmental Geochemistry, Institute of Geochemistry, Chinese Academy of Sciences, Guiyang 550081, China

**Keywords:** small gap disturbance, plant species richness, soil topography gradient, subtropical secondary forest

## Abstract

*Background*: The secondary forests have become the major forest type worldwide, and forest gap was also a common small disturbance in secondary forests. We aimed to analyze the effects of small gap disturbance on the plant species richness of subtropical secondary forest with natural regeneration barriers and examine the relationship between soil topography and plant species in a subtropical Rhododendron secondary forest of the Baili Rhododendron National Nature Reserve. *Methods*: The major plant species and soil topography gradient factors of the small gaps and closed canopy (control group) were analyzed using two-way ANOVA, multivariate permutational analysis of variance, nonmetric multi-dimensional scaling, random forest, canonical correspondence analysis, redundancy analysis, and a generalized linear model. *Results*: Small gaps had significant impact on the distribution of soil available potassium (AK), organic carbon to total phosphorus (C/P) ratio rather than slope position for soil pH and calcium (Ca) under closed canopy. Soil pH and AK followed by total phosphorus (TP) were the most important variables explaining the spatial distributions of soil properties in both habitats. Determining the spatial distribution of individual woody plant species were soil pH in small gaps, instead of lower altitude, TP, total potassium (TK) and sodium (Na) concentrations for both habitats. Moreover, Ericaceae and Fagaceae were strongly associated with pH in the small gaps. However, there was soil Na for the herbaceous plant in the closed canopy. The species richness of woody plant species in small gaps was affected significantly by pH, soil water content (SWC), and TK, instead of soil organic carbon (SOC), SWC and C/P ratio in both habitats. *Conclusions*: Small gaps were not always significantly improved the composition of soil nutrients, but provided a good microenvironment for plant growth, species richness of major woody plant differed between habitats.

## 1. Introduction

Forest canopy gaps play an important role in forest stand dynamics, affecting the patterns of forest stand development and changing species composition of forest succession process [1]. Forest gaps could help to preserve biodiversity, influencing nutrient cycles, and maintaining the complex forest stand structure of late-successional forests [2,3]. Gaps increase habitat diversity, structural complexity, species diversity, and improve levels of seedling survival [4,5]. The formation of canopy gap changes the original growth environment of plants and may create a suitable condition for their growth. Previous studies have shown that forest light conditions, air temperature, soil temperature, and moisture were changed after gap formation [1,6], Among these environmental factors, light plays a key role in plant growth during gap filling [6]. Gap size affects the distribution of light on the surface of tropical and temperate forest gaps, as gap size increases, light radiation into the forest gap increases [7]. Gaps in mature forests profoundly affect species composition and stand structure in tropical, temperate, and boreal forest types [4]. The canopy gaps may accelerate carbon (C) turnover and nutrient cycling in the forest successional processes [8] and decrease the nitrogen (N) and phosphorus (P) released by litters in warm subtropical forest [9], which may provide suitable nutrient environment for forest plant growth.

The secondary forest has become the major forest type worldwide [10]. In tropical regions, secondary forest of many countries cover more area than old-growth forest [11]. Secondary forest has an important impact on global ecosystem balance, it is an important carbon sink [12], and there was higher soil organic carbon (SOC) content in natural secondary forest [13]. Moreover, some studies have reported relationship between secondary forest species and their environmental factors. For example, the change of soil microbial biomass is related to the quantity and quality of soil organic matter during the succession of secondary forest [14]. The variation of herbaceous population size is affected by the size and number of high-light horizontal patches in pine-hardwood secondary forests [15]. In addition, Chen et al. [16] applied remote-sensing technology to study the investigation and monitoring of Rhododendron germplasm in Rhododendron secondary forest of Baili Rhododendron. A study of the hybridization behavior of *Rhododendron delavayi* Franch, *R. decorum* Franch. and *R. irroratum* Franch. in the growth process of subtropical Rhododendron secondary forest was completed by Zhang et al. [17] in same study area. However, few reports on the relationship between soil properties and plant species in subtropical evergreen broad-leaved forest gaps, especially in Rhododendron secondary forest.

The forest gaps created by natural processes are generally small and ephemeral, the importance of small gap-forming disturbances has emerged as a common theme in research on forests dynamics and natural regeneration in a variety of forests worldwide [18,19]. Previous research showed that both soil processes and properties are affected by gap size and these variations in the soil properties of forest gap play a vital role in seed germination and the establishment and recruitment of seedlings, which affect regeneration of different plant species [20,21,22,23]. Although the importance of gaps in forest succession is well-recognized the effects of small gap formation on soil properties and plant species in Rhododendron secondary forest of subtropical evergreen broad-leaved forest are not well-understood.

Some studies found that the distribution of soil property was related to the topography of different forest ecosystems [24,25]. Soil potassium (K) and calcium (Ca) contents in gap centers increase with an increase in elevation, but the pH increases and Al decreases in closed forest canopy [26]. Moreover, soil topography and forest gaps are the main factors affecting distribution of plant species [27,28]. At present, only a few rhododendron seeds germinate under a closed canopy in the Baili Rhododendron National Nature Reserve, and many rhododendrons (i.e., *R. delavayi* and *R. agastum* Balf.f.et W.W.Smith) have died gradually after suffering from pests and diseases. However, sporadic and well-established rhododendron seedlings and saplings have been found in small gaps. Previous studies have shown that lack of understory species beneath *R. formosanum* Hemsl. canopy was due to allelopathic effects [29], but it is unknown whether small canopy gaps of the subtropical Rhododendron secondary forest cause differences in spatial distribution of soil properties and plant species richness, and it remains unknown how soil topography gradients affect the spatial distribution of plant species and relationship between major plant species richness and soil topography gradients. Answering these questions may help us to understand the mechanism of natural regeneration barriers of closed canopy Rhododendron communities. It may also assist in maintaining species richness and management activities of subtropical Rhododendron secondary forests. The objectives of this study were to: (1) observe the distribution difference in soil properties (including different slope position soil properties) and plant species richness between small gap and closed canopy. (2) identify the most important soil property covariates and reveal the relationship between individual plant species and soil topography gradient factors in small gap and closed canopy. (3) determine the relationship between major plant species richness and soil topography gradient factors (i.e., topography gradients and soil properties) in both habitats, and evaluate the relationship between soil topography gradient factors and plant species richness of small gap.

## 2. Materials and Methods

### 2.1. Study Site

**Figure 1:**
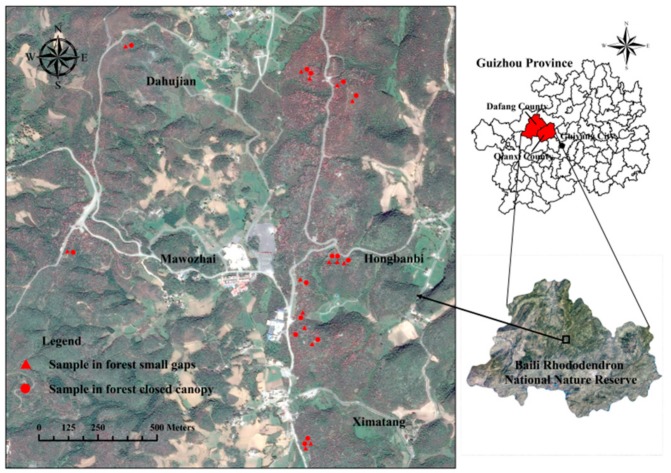
Spatial distribution of the sampling sites.

Baili Rhododendron National Nature Reserve (E 105°52′–106°03′, N 27°10′–27°20′) is located in northwest Guizhou Province, Southwest China (Figure 1). It is the largest Rhododendron secondary forest in the same latitude and middle-low altitude areas discovered. Rhododendron forest is a valuable and rare plant in southwestern forest region of China that distributes sporadically in mountain and hilly evergreen broad-leaved forests of Guizhou, Yunnan and Sichuan Province [17]. Compared with other places, the Rhododendrons are concentrated in the research area of Baili Rhododendron National Nature Reserve, Rhododendron secondary forest is a typical subtropical secondary forest. The evergreen broad-leaved forests were cleared due to human activity in 1950s, which led to the dominance of rhododendron shrubs in the secondary forest [17]. The forest community is formed by dominant rhododendron species (i.e., *R. delavayi*, *R. agastum*, and *R. irroratum*). Besides, *R. simsii* Planch, *Cyclobalanopsis glauca* (syn. *Quercus glauca*), *Aralia chinensis* L., *Eurya japonica* Thunb., *Fargesia spathacea* Franch, Pteridophyta and Bryophyta, among others, are common plant species. The soil types are mainly siliceous yellow soil and coal seam soil. According to the results of field investigations, there are thick and difficult-to-decompose litter (i.e., leaf litter) cover under the Rhododendron secondary forest, and a large number of small gaps in the forest canopy. The main reasons for the small gap formation are human disturbance and natural disturbance regime.

### 2.2. Sampling Points Setting

**Figure 2:**
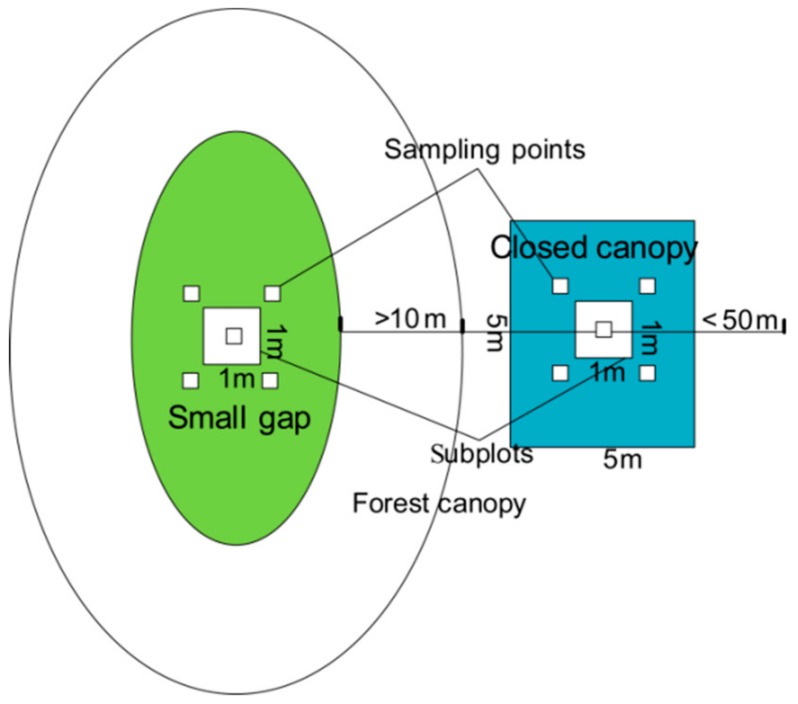
Experimental design of sample plots and sampling points between small gap and closed canopy.

Sample survey time was from November to December 2016. Thirty plots of forest small gaps (G, *n* = 15) and closed canopy (CC, size: 5 × 5 m^2^; *n* = 15) were used to collect data on both soil topography gradient factors and major plant species (Figure 1). The small gap was defined as an area where the canopy was opened by a gap size within 15–30 m^2^, and plots were randomly selected. The closed canopy was defined as an area where crown cover is >80%, and trees forming the crown cover are >3 m tall. Closed canopy plots were located in a forest canopy area 10 m < size < 50 m away from the edge of the forest small gaps, surrounding the forest small gaps without orientation restrictions. A 1-m^2^ subplot was established in the center of each gap or closed canopy, which was at least 0.8 m from any stumps to avoid disturbing dead roots. Soil samples were taken from five sampling points, one at the center of the 1-m^2^ subplot and four at the outside of four corners, five samples were mixed to obtain a representative sample for each plot (Figure 2). Soil cores of one depth (0–10 cm) were randomly collected in each subplot, and litter and organic debris were removed. Fifteen closed canopy soil samples among all subplots were taken as a control group to compare the differences between small gaps and closed canopies. The soil samples were blended before sealing in a plastic bag and transporting them back to the laboratory to determine the soil properties. The plant species were recorded in each plot of small gap and 5 × 5 m^2^ closed canopy.

Plant species in small gap and closed canopy plots are recorded (Table 1). There were 13 major woody plant species distributing in the small gap, and 8 woody plant species (except for *Fargesia spathacea*) distributing in the closed canopy. In this study, the slope was the angle between the litter surface of the sampling site and the horizontal surface, and slope of each plot is collected within a 1-m^2^ subplot. The slope of all plots were between 0 and 50°. The slope position was also divided into three groups (i.e., downhill position, mid-slope position, and uphill position), the number of sample plots are 3,5,7 for small gaps and 3,7,5 for closed canopy, respectively. The plots spatial distribution was designed by slope position. All plots were located between 1590–1776 m. The altitude, latitude, longitude, and slope of each sampling site were recorded using a hand-held Global Positioning System device.

### 2.3. Soil Sample Pretreatment and Measurement

The collected soil samples were mixed, air-dried naturally, the stones and plant rhizomes were picked out, and they were passed through a 2-mm sieve to separate fine earth and coarse soil fractions in the laboratory, and placed in a sealed bag to measure soil properties. Soil pH was measured in a 1:2.5 soil-to-water suspension ratio; Soil organic carbon (SOC) was measured using the K_2_Cr_2_O_7_-capacitance method; Total nitrogen (TN) was measured using the micro Kjeldahl method [30], total phosphorus (TP) was measured using NaOH fusion and Mo-Sb colorimetric procedures, and hydrolysable nitrogen (HN) in the soil was determined using the diffusion-absorption method. Available potassium (AK) was extracted with neutral ammonium acetate and was measured using flame photometry; Available phosphorus (AP) was extracted with NaHCO_3_ solution and its contents were determined using the Mo-Sb colorimetric method [31]. Soil water content (SWC) was determined gravimetrically by drying 52–126 g of field moist soil sample at 105 °C for 24 h. The soil samples were acid digested with a 2:2:1 mixture of HNO_3_-HF-HClO_4_ [32], followed by 5300 V inductively coupled plasma optical emission spectrometry (Perkin Elmer, Waltham, MA, USA) to determine the concentrations of total potassium (TK), calcium (Ca), magnesium (Mg) and sodium (Na). These determinations were also performed in blank parallel experiments.

### 2.4. Data Analysis

The soil properties data (including carbon/total nitrogen/total phosphorus (C/N/P) ratio) of forest small gaps and closed canopy with different slope positions were used to test whether there were significant differences between the two groups (small gaps and closed canopy), Pearson’s correlation analysis was performed to analyze the correlations between plant species richness and soil soil-topography gradient factors in both habitats. Species richness was the total number of species per plot, which was an important characteristic of a community and index for examining the species diversity of a plant community [33]. Plant species richness was the total number of major plant species observed in per plot in this study. Statistical analysis to evaluate the effects of the gap type and slope positions on the measured soil property parameters was conducted by two-way ANOVA following the general linear model (GLM) procedure using the Bonferroni pairwise multiple comparison test at alpha level of 5%. These analysis processes were performed by using the SPSS 24.0 for Windows program (SPSS Inc., Chicago, IL, USA). The permutational multivariate analyses of variance (PERMANOVA) analyzed soil properties differences and plant species richness differences between groups. The number of permutations was set at 999 (Significantly different at *p* ≤ 0.05). Then soil properties and plant species richness were analyzed and plotted using nonmetric multi-dimensional scaling (NMDS), The stress values below 0.2 are regarded as potentially useful, whereas values below 0.1 are regarded as good ordinations [34]. This would test whether there are differences between groups broadly across all soil variables, which was performed by using the package “vegan”, “ggrepel” and “ggplot2” on R statistical platform (v 3.5.0) (R Foundation for Statistical Computing, Vienna, Austria).

A canonical correspondence analysis (CCA) was used for ordination to analyze the variations in the individual plant species and their relationships with soil topography gradient variables. The statistical data of woody and Herbaceous plants were used for this analysis. The slope position was divided into numerical grades, such as downhill position 1, mid-slope position 2, and uphill position 3, and other data adopted the actual measured values. CCA was performed using the function of “vegan” and “ggplot2” package on R statistical platform (v 3.5.0). The soil topography gradient variables, which have a significant impact on individual plant species in CCA and significantly correlation with plant species richness in Pearson’s correlation analysis, were used to evaluate the relationship between soil topography gradient factors and plant species richness of small gap by employing the generalized linear models (GLMs) in the SPSS 24.0 for Windows program. Origin pro (version 8.5.1) (Origin Lab Corp., Northampton, MA, USA) and ArcGIS 10.3 software (Environmental Systems Research Institute Inc., Redlands, CA, USA) were used for drawings.

Random Forest (RF) was used to identify the most important soil property covariates in the small gaps and closed canopy using the “random forest” package in R version 3.5.0. The mean decrease in accuracy is based on the measure of the importance of the substitution variable, which turns the value of a variable into a random number and measures the degree to which the RF prediction accuracy decreases. The mean decrease in Gini compares the importance of variables by calculating the effect of each variable on the heterogeneity of observations at each node of the classification tree. A variable with a higher importance score compared to other variables indicates that the variable is important for classification [35]. In the process of running the model, the small gaps and closed canopy soil variables were response variables, which were divided into two groups.

## 3. Results

### 3.1. Soil Properties in the Small Gaps at Diffferent Slope Positions

Soil AK content (F_1,24_ = 12.29, *p* = 0.02), C/P ratio (F_1,24_ = 5.60, *p* = 0.03) were significantly affected by gap types, soil pH (F_2,24_ = 4.85, *p* = 0.02) and Ca content (F_2,24_ = 6.09, *p* = 0.007) were significantly affected by slope position, whereas the interaction of gap types and slope position did not significantly affect the spatial distribution of those soil properties (Table 2). Other soil properties were also not significantly affected by gap types, slope position and their interaction. There were significant differences for some topsoil properties between small gap and closed canopy (Table 2). Soil AK content in the downhill position and mid-slope position gap topsoil were significantly higher than those of the closed canopy, respectively. Soil C/P ratio in different slope positions were lower than those in the closed canopy. Soil pH and Ca content in topsoil of the downhill slope position closed canopy were also significantly higher than those in the mid-slope position closed canopy; no differences were observed in the other soil properties (*p* > 0.05). These results also demonstrated that the small gap topsoil of downhill position and mid-slope position had sufficient soil AK to promote their plant growth.

### 3.2. Soil Properties Differences Between Small Gaps and Closed Canopy

**Figure 3:**
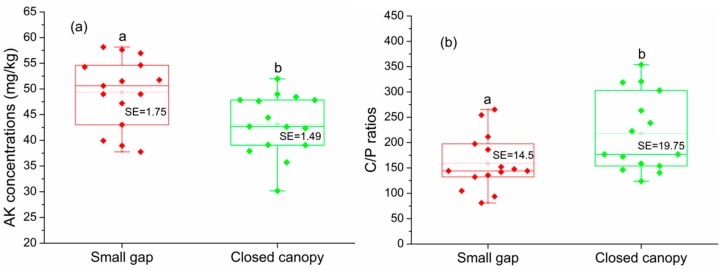
The soil AK content and C/P ratio of small gaps and closed canopy (*n* = 15:15). (**a**): AK content of small gaps and closed canopy; (**b**): C/P ratio of small gaps and closed canopy. Paired groups marked by letters a and b or A and B in box plots are significantly difference (based on two-sided independent *t*-tests, *p* ≤ 0.05).

**Figure 4:**
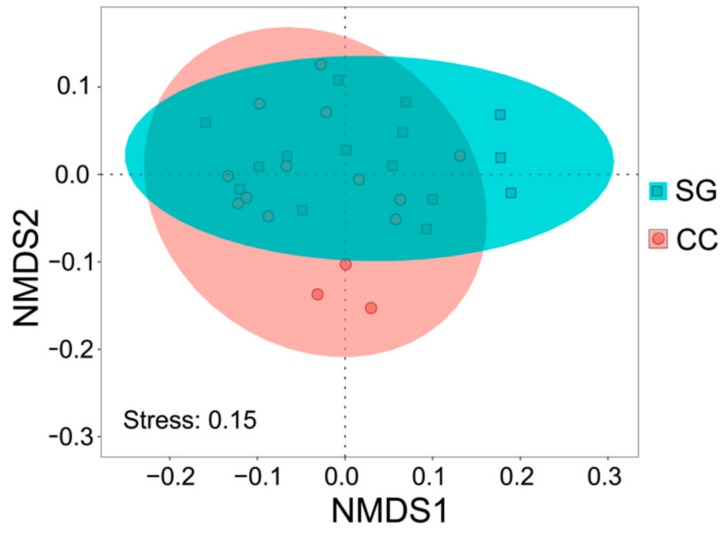
Nonmetric multi-dimensional scaling (NMDS) of soil properties (hellinger-transformed soil property data; distance = “bary”). SG: small gaps (*n* = 15); CC: closed canopy (*n* = 15).

The *t*-test results indicated that soil AK (*t* = 2.87, *p* = 0.008) in the small gap topsoil was significantly higher than that in the closed canopy, while the C/P ratio (*t* = −2.52, *p* = 0.018) was significantly lower than that of the closed canopy (Figure 3a,b); no significant differences were detected for the other soil properties (*p* > 0.05). We also did not detect significant difference on soil properties between the small gaps and closed canopy (PERMANOVA: *r*^2^ = 0.06, *F* = 2.29, *p* = 0.07 > 0.05). NMDS analysis for soil properties provides a fairly good representation of the small gaps and closed canopy topsoil (Figure 4, stress = 0.15), most of plots also tend to separate according to sampling site. Nevertheless, the two group soil properties of the small gap and closed canopy were not well separated on the NMDS scatterplot, the soil properties of small gaps and closed canopy did not show significant differences.

### 3.3. Importance of Soil Properties in the Subtropical Rhododendron Secondary Forest

**Figure 5:**
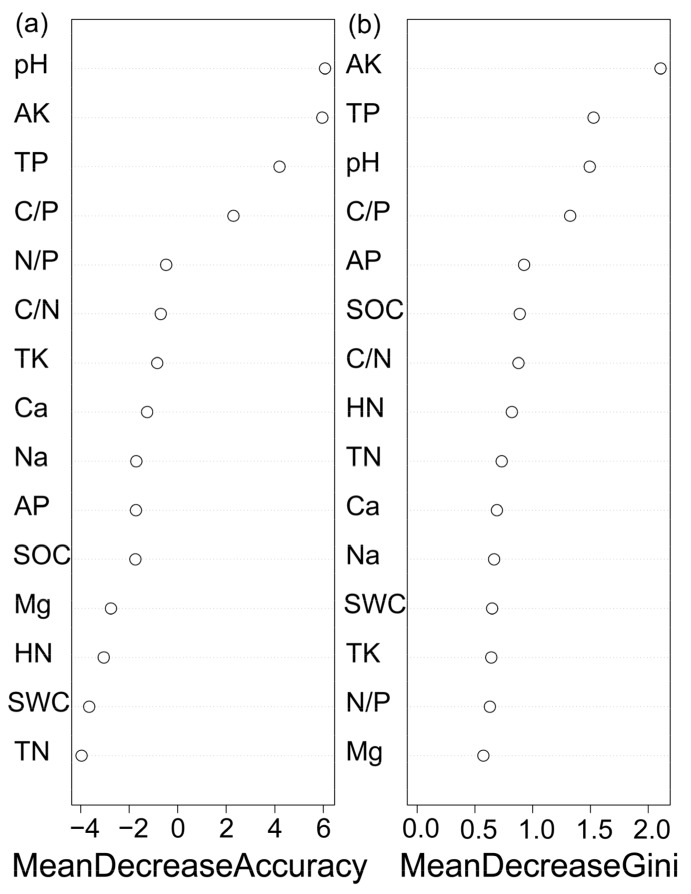
Ordination of variable importance derived from the Random forest (RF) models for the soil property variables. (**a**) Mean Decrease in Accuracy, (**b**) Mean Decrease in Gini.

According to the variable importance measurements, the soil pH, AK, and TP were the most important variables for small gaps and closed canopy. The variables that increased model accuracy the most were pH and AK, followed by and TP (Figure 5a). AK played the most effective role producing high homogeneity in the descendent nodes (Figure 5b). These results indicate that pH and AK had the most important impact on the surface soil properties in the subtropical Rhododendron secondary forest, followed by TP.

### 3.4. Plant Species Richness and Composition of the Small Gaps and Closed Canopy

**Figure 6:**
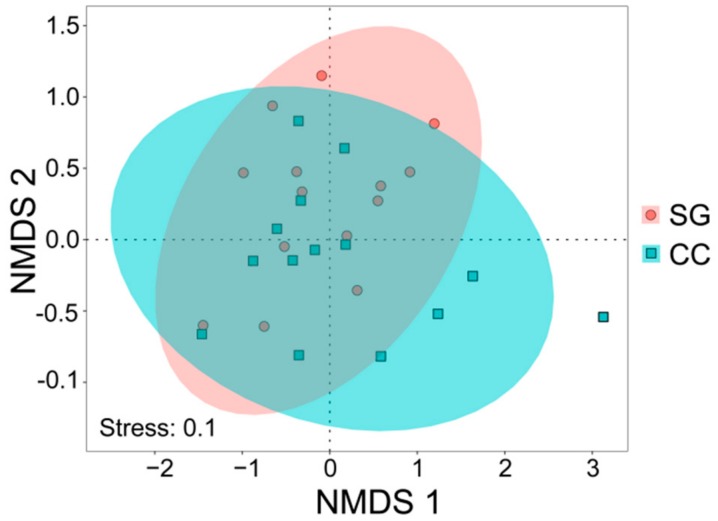
Nonmetric multi-dimensional scaling (NMDS) of plant species (Log (number of individuals +1) was used to transform plant species data; distance = “jaccard”). SG: small gaps (*n* = 15); CC: closed canopy (*n* = 14, there is a plot missing the main plant species).

Small gap formation changes the distribution characteristics of main plant species in Rhododendron secondary forests. Although the NMDS analysis for plant species provided a fairly good representation of the small gaps and closed canopy (Figure 6, stress = 0.1), and most of plots also tended to separate according to sampling site, the plant species of small gaps and closed canopy did not show significant differences. A comparison of plant species using the PERMANOVA (*p* ≤ 0.05) also found no significant difference between two habitats (*r*^2^ = 0.04, *F* = 1.11, *p* = 0.38). There were higher plant species composition similarity between two habitats. However, we did detect significant difference on plant species richness between small gaps and closed canopy (*r*^2^ = 0.19, *F* = 6.24, *p* = 0.02). Compared with the closed canopy, the small gap had higher plant species richness.

### 3.5. Relationship between Individual Plant Species and Soil topography Gradient Factor

**Figure 7:**
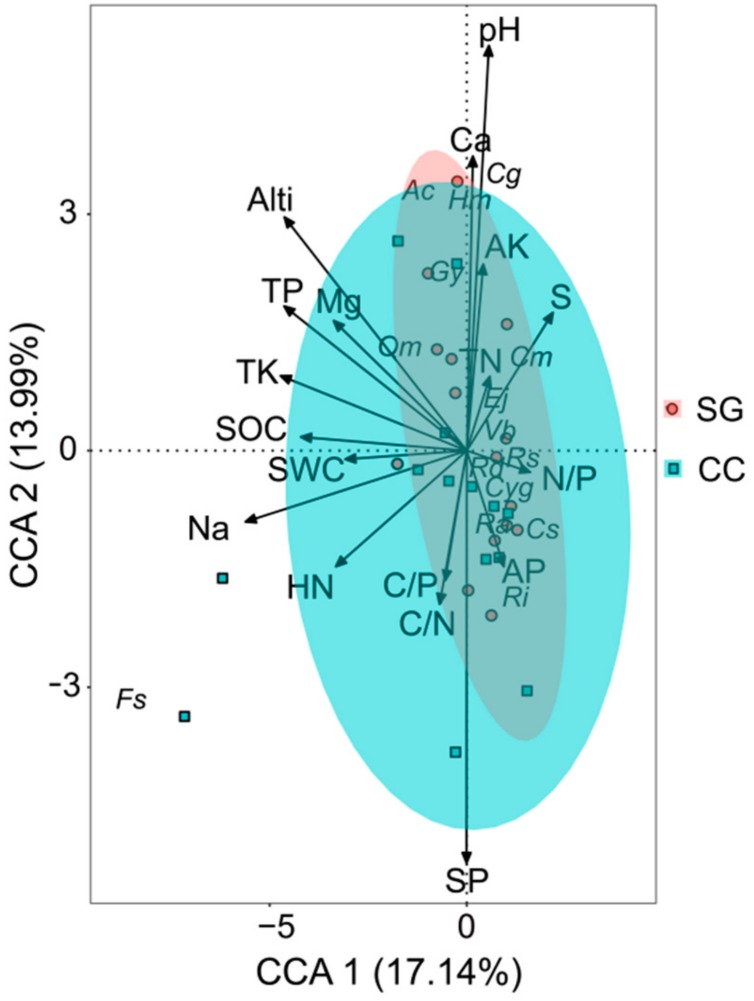
Ordination of the main plant species and soil topography gradient variables of the small gap and closed canopy in the canonical correspondence analysis (CCA). Log10 (number of individuals +1) was used to transform plant species data. SG: small gaps (*n* = 15), CC: closed canopy (*n* = 14). Significantly different at *p* ≤ 0.001 and *p* ≤ 0.05. Alti: altitude, S: slope, SP: slope position. *Cyg-Cyclobalanopsis glauca*, *Hm-Hypericum monogynum*, *Cm-Castanea mollissima*, *Gy-Gaultheria yunnanensis*, *Vb-Vaccinium bracteatum*, *Qm-Quercus michauxii*, *Rd-Rhododendron delavayi*, *Ri-Rhododendron irroratum*, *Ra-Rhododendron agastum*, *Rs-Rhododendron simsii*, *Cs-Castanea sequinii*, *Ej-Eurya japonica*, *Cg-Cinnamomum glanduliferum*, *Ac-Aralia chinensis*, *Fs-Fargesia spathacea*.

Although the first two axes of the CCA cumulatively only explained 31.13% of the variance in the species-environment relationship, the spatial distribution of individual plant species in relation to environmental variables. Permutation test demonstrated that the relationship between individual plant species and soil topography gradient variables is highly significant (*F* = 1.61, *p* = 0.005; 999 permutations). The first axis had stronger negative correlations with altitude (*r*^2^ = 0.27, *p* = 0.006), Na (*r*^2^ = 0.28, *p* = 0.02), TK (*r*^2^ = 0.21, *p* = 0.04), TP (*r*^2^ = 0.22, *p* = 0.02). Similarly, the second axis had a stronger negative correlation with slope position (*r*^2^ = 0.25, *p* = 0.01), and positive correlation with pH (*r*^2^ = 0.24, *p* = 0.03) (Figure 7). These soil topographic factors also had greater impact on plant species distribution, since they had a bigger *r*^2^ value. Comparing with closed canopy sampling plots, small gap samples plots tend to aggregate distribution with an elliptical on both sides of the second axis (Figure 7).

The woody plant species that were positively and closely associated with pH included *C. glanduliferum*, *H. monogynum*, *G. yunnanensis*, *A. chinensis*. The distribution of these woody plants was primarily affected by the pH value. Except for *A. chinensis*, other three woody plants grow in small forest gaps. In contrast, *R. irroratum*, *R. agastum*, *R. simsii*, *R. delavayi*, *C. glauca* and *C. sequinii* were negatively correlated with pH, altitude, TP, TK and Na (Figure 7). These results suggest that pH, altitude, TP, TK and Na are important soil topography gradient factors that significantly contribute to determine the spatial distribution of small gap woody plant species, especially in the distribution of small gap woody plants *R. simsii* and *C. sequinii*. The distribution of herbaceous plant *F. spathacea* is prominently affected by higher soil Na concentration in closed canopy. Ericaceae and Fagaceae species were more abundant in the lower pH, altitude, slope position, Na, TP and TK value areas, where soil AP and N/P ratio were high.

### 3.6. Relationship Between Plant Species Richness and Soil Topography Gradient Factors

There were many significant correlations between plant species richness and soil soil-topography gradient factors. The plant species richness was significantly correlated with the soil SOC (*r* = −0.453, *p* ≤ 0.05), SWC (*r* = −0.486, *p* ≤ 0.01) and C/P ratio (*r* = −412, *p* ≤ 0.05) in the small gaps and closed canopy, respectively. Based on the results from CCA and Pearson’s correlation analysis, the small gap soil topography gradient variables, which reached a significant level (*p* ≤ 0.05) of statistical analysis, were used to perform generalized linear models (GLMs) with plant species richness of small gap (Table 3). The GLMs output shows that the species richness of main woody plant species in forest small gaps is affected by native soil topography gradient factors (Table 3). In particular, pH, SWC, and TK are the most significant predictors in explaining plant species richness of small gap. The increase or decrease of these soil and topographic factors may affect the distribution of woody plant species in forest small gaps.

During modeling, the GML models were evaluated by comparing the fitting model with the intercept model according to the Omnibus test (likelihood ratio test, *p* ≤ 0.05). The evaluation results show that the fitting models of plant species richness (χ^2^(7) = 21.67, *p* = 0.003), are significantly different from their intercept models, respectively. Furthermore, multi-collinearity was diagnosed by examining the variance inflation factors (VIFs) for the predictors, The VIFs of the predictors was well below the rule-of-thumb cut-off of 10 [36]. According to CCA results, soil Na (VIF: 11.97) and TP (VIF: 16.06) with VIFs value >10 were stepwise removed before modeling because they had less influence on the distribution of small gap plant species. Thus, GMLs effectively reflected the effect of predictors (soil topographic factors) on species richness and their internal relationship in small gap.

## 4. Discussion

### 4.1. Effects of Small Gap Disturbance on Soil Properties in Different Topographies of the Subtropical Rhododendron Secondary Forest

Forest gap did not always promote nutrient cycling and changed nutrient composition of the forest topsoil, especially in the small gap of the Rhododendron secondary forest. There are significant differences for some topsoil properties between small gaps and closed canopy. Except for the influence of small gaps, soil properties were also affected by the slope position. After the formation of small gaps, the distribution of soil AK and C/P ratio in Rhododendron secondary forest was significantly affected. However, slope position was the main influencing factor for the distribution of soil pH and Ca under closed canopy. Soil AK content in the downhill and mid-slope position gap topsoil were significantly higher than those of the closed canopy, respectively, while the C/P ratio of different slope position was lower than that of the closed canopy. Forest gap improved the rate of soil organic matter decomposition and mineralization, leading to increased nutrient contents [37], which may cause an increase of soil AK content at the mid-slope and downhill slope positions. Mean soil SOC content in the mid-slope position small gap (102.12 ± 20.35 g/kg) was 10.01% lower than that in the closed canopy (113.48 ± 14.14 g/kg). Moreover, soil SOC content of downhill position small gap (80.59 ± 6.06 g/kg) was 51.68% lower than that in the closed canopy (130.41 ± 63.01 g/kg). In this study, TP content of different slope position small gaps showed increasing trend, especially in the mid-slope position. A study showed soil TP released by litters in canopy gap of warm subtropical forests [9], which may cause a lower the C/P ratio in the small gap. Compared with the higher temperature and moisture of summit position gap may also stimulate soil nutrition leaching [38], moderating soil temperature, moisture and leaching of medium and downhill position small gap may accelerate carbon turnover, litter decomposition and soil nutrition accumulation. The C/P ratio depending on local compositions of soil and litter, carbon and phosphorus was released during litter decomposition [39], following gap creation, the litterfall was reduced, resulting in the accumulation of carbon decreased [8], thus potentially reducing the small gap soil C/P ratio.

The pH value of the topsoil in the mid-slope position small gap was higher than that in the closed canopy, which may increase soil potassium availability of the small gaps, because higher soil pH could improve the availability of nutrients [40]. Soil pH of the downhill position had a weaker effect on the AK content because where the small gap with a lower pH value. Soil pH and Ca content in topsoil of the downhill position closed canopy were significantly higher than those in the mid-slope position closed canopy. Similarly, Previous studies have shown that slope position was closely related to the spatial distribution of soil property, and downhill position also had higher soil Ca content [24], mainly due to the weakening of the leaching and the increase of soil nutrient accumulation [41], which may lead to increase of pH and Ca content in downhill position closed canopy. Additionally, the increase of pH and Ca content in closed canopy topsoil may be affected by other factors (e.g., degree of canopy closure, soil development processes, accumulation of organic matter and litter).

### 4.2. Effects of Small Canopy Gap on Soil Properties in the Subtropical Rhododendron Secondary Forest

Small-scale forest gap significantly increased soil AK content and reduced the soil C/P ratio. Similarly, soil AK content in small gaps is higher than that of the closed canopy in a *Castanopsis Kawakamii* Hayata natural forest [42]. The forest gaps may have improved the rate of soil organic matter decomposition and mineralization, as litter mineralization processes prevail during humification in the small gaps [21], leading to increased nutrient levels [37]. The mean SOC content of the small gap topsoil (105.55 ± 10.77 g/kg) was lower than that of the closed canopy (116.39 ± 14.10 g/kg) by 9.31%, Soil AK content in the small gaps was 15.44% higher than that of the closed canopy, and TP content (0.69 ± 0.05 g/kg) was 25.45% higher than that of the closed canopy (0.55 ± 0.05 g/kg). The field investigations revealed that the accumulation and decomposition of litter were limited in the closed canopy of the Rhododendron secondary forest. A study has reported that the solar radiation, temperature, and moisture conditions generally change after gap formation [22], which may accelerate decomposition of litter, and the small canopy gaps may lead to enhanced leaching in a Rhododendron secondary forest, resulting in reduced accumulation of SOC and other nutrients in the topsoil. Nevertheless, there were not significant differences in soil properties between the small gaps and closed canopy. pH and AK followed by TP were the most important variables explaining the spatial distribution of the soil properties in small gaps and closed canopy. The mean pH value (4.45 ± 0.21) in the gaps was 4.46% higher than that in the closed canopy (4.26 ± 0.14), potentially increasing AP and AK contents in the gap topsoil, because lower soil pH and limit the availability of nutrients such as phosphorus and potassium, elevating pH increased soil phosphorus and potassium availability [43]. Soil pH also affected the form and availability of soil organic matter and its associated processes, including microorganism activities and biological growth [22]. Moreover, soil pH, AK, and TP exhibited a highly significant relationship with other soil properties in Rhododendron secondary forest topsoil in our study.

The differences in allelopathy of rhododendrons cannot be ignored after gap formation. For example, *R. formosanuin* flowers, leaves, litter and organic matter contain allelochemicals, especially phenolic acid, including p-hydroxybenzoic acid, protocatechuic acid, ferulic acid syringic acid and vanitlic acid are major allelochemicals released by *R. formosanuin* leaves [29]. The litter (leaf litter) decomposition at the soil surface gradually changes the soil acidity and phenolic acid content [44], but the limited decomposition of rhododendron litterfall increased soil acidity (mean pH, 4.26) in closed canopy. The acidic soils found in the rhododendron rhizosphere (e.g., *R. formosanum*) could be attributed to the accumulation of phenolic compounds from metabolites and compounds released from the decomposition of organic matter in the soil [45]. The micro-environment formed by gaps promoted the decomposition of litter, resulting in higher phenolic acid content in the small gap topsoil than in the closed canopy, and soil acidity decreased (mean pH, 4.45), while phenolic acid was a strong complexing agent for heavy metals (i.e., manganese, ferrum, and aluminum), but their synergistic effect caused sedimentation or adsorption of dissolved heavy metals and nutrients (i.e., organic matter, nitrogen and phosphorus) in the small gap topsoil [46]. Allelopathy of rhododendrons restricted nutrient absorption by rhododendron seedlings, which was not conducive to growth and regeneration of rhododendrons in small gaps. In contrast, the low soil pH value may have decreased hydrogen bonding between the hydroxyl groups of the phenolic compounds and the soil particles, which facilitates desorption of phenolic acid from the topsoil [47]. Therefore, the phosphates in the closed canopy topsoil and soil potassium concentrations in the gaps increased, respectively, and ericoid mycorrhizal fungi may increase nutrient uptake in the roots of rhododendron species [48], which possibly promoted rhododendrons growth in the small gaps.

The effect of forest gaps on soil properties is a complex process. The variations in soil properties are also related to winter and growing season, litter thickness and its decomposition rate, the return of root biomass, and other factors [49,50]. Previous studies have shown that soil properties are significantly affected by forest gaps, the forest gap in alpine forests slows the releases of elements such as copper and zinc in foliar litter during forest regeneration in azalea (*R. lapponicum* (L.) Wahlenb) [50]. Additionally, the largest populations of bacteria and fungi and the greatest amount of microbial biomass contributes to a more balanced and rapid turnover of organic matter and nutrients in small gaps [51]. The effect of small gap disturbances on soil properties may also be affected by the size of the trunk disturbance, forest height, soil parent material, and soil weathering environment. The ecological function of small gap needs further study.

### 4.3. Plant Species Richness and Composition

The effect of small gaps was also reflected in the resulting plant species richness and composition in subtropical Rhododendron secondary forests. Results of PERMANOVA clearly suggested that the plant species composition of small gaps were not significant difference compared with the sample plots of the closed canopy, but the differences of plant species richness was significant in two habitats. Small gap could create higher richness compared to closed canopy. In this study, plant species richness of the small gap (3.60 ± 0.41) was 62.90% higher than that of the closed canopy (2.21 ± 0.30). A study of plants composition and richness at subtropical forests between gaps and closed canopy patches also found similar conclusions [52], more importantly, reaffirms the importance of gap disturbance in maintaining tree richness in subtropical forests. However, compared with similar small gap (mean gap size 10 m^2^ and 30 m^2^) studies, there were very high plant community similarity (>90%) between gaps and non-gaps of subtropical forest [53], because the small gaps play a neutral role in understory light environment, thus provide had a neutral role in the plant richness. Small gap formation may improve closed canopy environment factors (e.g., light) for seedling establishment and growth in subtropical Rhododendron secondary forest. In addition, the small gaps favored the recruitment of a suite of species quite distinct from that found under a closed canopy [54], which possibly increased richness of small gap plant species.

### 4.4. Relationship Between Individual Plants Species and Soil topography Gradient Factors

The CCA analysis indicated that soil topography gradient variables (i.e., altitude, pH, TP, TK and Na) were important to determine the spatial distribution of plant species in small gaps and closed canopy. The relationship between individual plant species and soil topography gradient variables was highly significant. The distribution of woody plant *C. glanduliferum*, *H. monogynum* and *G. yunnanensis* were positively and closely associated with pH in small gaps, whereas gap woody plant *R. simsii* and *C. sequinii* were negatively correlated with altitude, pH, TP, TK and Na. The woody plant *R. irroratum*, *R. agastum*, *R. delavayi* and *C. glauca* were also negatively correlated with pH, altitude, TP, TK and Na in both habitats. Ericaceae and Fagaceae were more abundant in the areas where environmental factors were lower pH, altitude, slope position, Na, SOC, TP and TK. Similarly, growth of rhododendron species had found a good relationship with pH and elevation in Gaoligong Nature Reserve of China and in the Kanchenjunga Conservation Area and Jaljale Himal region of Nepal [55,56], previous study showed that the mean pH was 5.53 in *R. ferrugineum* L. forest, while at *R. hirsutum* L. forest it was 7.26 [57]. The altitude could cause a strong division of soil and light resources, affecting the redistribution of temperature and precipitation [26], leading to differences in species distributions. The distribution of herbaceous plant *F. spathacea* was prominently affected by higher soil Na concentration in closed canopy. A previous study indicated that soil salinity was one of the important factors affecting vegetation distribution and plant growth. Our results showed that the mean content of soil Na of closed canopy (1.69 ± 0.45 g/kg) was higher (19.01%) than that of the small gap (1.42 ± 0.36 g/kg). Increased soil salinity and weakened nutrient cycling may contribute to reduced plant species under the canopy. Furthermore, Özcan and Gökbulak (2015) reported that the sodium concentration decreases as gap size increase [22]. The woody plant species are more abundant in the small gaps with a higher pH value and lower Na, TP, TK contents, pH value and slope position rather than mainly affected by soil pH, elevation, nitrogen, organic matter and soil moisture [55,56]. Furthermore, the species survey also indicated that the plant species were more abundant in small gaps. Similar results have been reported by Mcclure et al. [58] and Rao et al. [59] in their study of forest small gaps, respectively, and the plant species richness and abundance distribution are significantly correlated with the soil topography gradient in relative forest gap areas.

### 4.5. Plant Species Richness and Soil Topography Gradient Relationship

Correlation analysis revealed that SOC, SWC and C/P ratio were the major factors that determined the spatial distribution of woody plant species richness on both small gaps and closed canopy. Field investigations have found that small gaps promoted the decomposition of litter, previous studies by Scharenbroch and Bockheim [37] who reported that the forest gap improved the rate of soil organic matter decomposition and mineralization, leading to increased nutrient contents. The formation of the forest gap had changed the micro-environment conditions, especially light transmittance and soil water content, which increased with gap size [60], accelerated the decomposition and mineralization of aboveground rhododendron litter, and changed the nutrient composition between the closed canopy and small gap to help the plant seedlings establishment and growth. Therefore, SWC and SOC play vital role in the spatial distribution of species richness.

The small gap increased plant species richness in the late gap regeneration of the Rhododendron secondary forest. During the process of regeneration and restoration, the soil topography gradient has a potential but important impact on the plant species richness of small gaps. The GLMs output showed that the species richness of main woody plant species in forest small gaps is significantly affected by native soil topography gradient factors. The pH, SWC, and TK are the most significant predictors in explaining plant species richness of small gap woody plant species. The formation of forest gaps also created the heterogeneity of forest environment, which may cause differences in the distribution of the environmental factors. Soil pH could influence nutrient availability and assimilation, elevating pH increased soil phosphorus and potassium availability, thus affecting plant growth and development [43,61], the small gaps also provided a good growth and soil nutrient environment for the growth of plant species by regulating the evaporation of water. The closed canopies can intercept a large fraction of rainfall. However, gaps may permit more precipitation to reach the forest floor, finally change plant species richness.

Farther small forest gap studies cannot ignore the importance of active restoration (i.e., forestry treatments, tree planting), control and pairing experiments. Most forest gap studies are based on retrospective analysis of natural gaps and not controlled experiments, it is often not clear how growth and mortality of understory trees, affect stand development and the process of gap closure [4]. A previous study showed that the active restoration was more effective than the natural regeneration at recovering tropical forests [62], however, little is known about the two ways of forest restoration impact on the succession process of small forest gaps and their soil properties in subtropical Rhododendron secondary forests. The best treatments may be to perform pairing experiments at the same site to optimize the complexity of forest restoration [63], and apply it to the artificially assisted regeneration and management of subtropical secondary forests to restore and strengthen their ecological service functions.

## 5. Conclusions

Small gaps did not significantly improve the structural composition and accelerated cycling of surface soil nutrients, except for soil AK and C/P ratio. Although slope position had a significant impact on the distribution of soil pH and Ca under closed canopy, small gaps of different slope positions were also not obvious for improving the composition of soil nutrients. Soil pH and AK was the most important variable affecting the spatial distribution of soil properties, followed by TP.

Small gap significantly increased species richness of the subtropical Rhododendron secondary forest and provided a good topsoil microenvironment for plant growth. The important soil topography gradient factors determining the spatial distribution of individual woody plant species were soil pH in small gaps rather than lower altitude, TP, TK and Na concentrations for both habitats. Moreover, Ericaceae and Fagaceae were strongly associated with soil pH in small gaps. However, soil Na had the prominent effect on the herbaceous plant *F. spathacea* in closed canopy. The species richness of woody plant species of small gaps was affected significantly by pH, SWC, and TK, instead of SOC, SWC and C/P ratio in both habitats.

## Figures and Tables

**Table 1:** Ranking of major plant species abundance in small gaps and closed canopy.

Plant Type	Major Plant Species	Small Gaps	Closed Canopy
Woody	*Rhododendron delavayi* Franch	8	8
	*Rhododendron agastum* Balf.f.et W.W.Smith	8	6
	*Quercus michauxii* Nutt.	6	3
	*Cyclobalanopsis glauca* (syn. *Quercus glauca*)	5	3
	*Eurya japonica* Thunb.	4	3
	*Rhododendron irroratum* Franch	4	5
	*Rhododendron simsii* Planch	4	-
	*Castanea sequinii* Dode	3	-
	*Hypericum monogynum* L.	3	-
	*Gaultheria yunnanensis* (Franch.) Rehder	3	-
	*Cinnamomum glanduliferum* (Wall.) Meissn.	2	-
	*Vaccinium bracteatum* Thunb.	2	-
	*Castanea mollissima* Blume	2	-
	*Aralia chinensis* L.	-	2
Herbaceous	*Fargesia spathacea* Franch	-	2

“-”: The species was not found in the small gap or closed canopy plots. The frequency of main species appearing in the 15 same type plots ≥ 2 was regarded as a main species. During statistical plant species of small gap and closed canopy plots, one plant species in each plot is recorded by the values 0 and 1 (0 means no found in the plot, 1 means the species grows in the plot).

**Table 2:** Relationships between gap type and slope position and soil properties.

Gap Type	Slope Position	AK (mg/kg)	C/P	pH	Ca (g/kg)
Small gaps	Downhill	50.99 ± 2.06A	143.91 ± 26.97	4.9 ± 0.41	2.63 ± 1.05
	Mid-slope	53.74 ± 1.83a	141.28 ± 20.17	4.22 ± 0.21	1.20 ± 0.27
	Uphill	45.66 ± 2.90	172.52 ± 25.92	4.41 ± 0.16	1.51 ± 0.29
Closed canopy	Downhill	38.70 ± 4.27B	218.60 ± 69.43	5.19 ± 0.99a	2.77 ± 0.89a
	Mid-slope	44.30 ± 2.14b	217.44 ± 21.06	4.01 ± 0.08b	1.11 ± 0.15b
	Uphill	43.19 ± 2.22	218.45 ± 41.54	4.13 ± 0.04	1.53 ± 0.27
Gap type ^c^		*	*	NS	NS
Slope position ^c^		NS	NS	*	*
G × SP ^c^		NS	NS	NS	NS

Dependent variable: soil properties. NS, not significant (*p* > 0.05). ^c^: Significance level for two-way ANOVA with gap type and slope position as main effects. The results were expressed as the means ± SE. In the two-way ANOVA analysis, the abnormal value is replaced by the second maximum value in the same group. Paired groups marked by letters a and b or A and B in the same column are significantly difference, * *p* ≤ 0.05 significant level.

**Table 3:** Results of generalized linear models (GLMs) exploring factors in the effects of the small gap soil topography gradient factors on plant species richness.

Coefficient	B	SE	WCT (χ^2^)	P (>χ^2^)
Intercept	5.60	8.53	0.450	0.503
Alti	0.01	0.01	3.45	0.063
SP	−0.58	0.30	3.68	0.055
pH	−1.28	0.52	6.00	0.014 *
SWC	−0.21	0.04	28.81	<0.000 *
SOC	0.00	0.01	0.00	0.971
TK	−0.56	0.17	10.31	0.001 *
C/P	−0.01	0.01	1.24	0.265
Scale	0.56a	0.21	-	-

Significance: * *p* ≤ 0.05; “-” represents no numeric value, B: Coefficient value, SE: standard error, a: maximum likelihood estimation, Alti: altitude, S: slope, SP: slope position. Distribution of species richness data of small gap (*n* = 15) was normally distributed with the Kolmogorov-Smirnov test (*p* > 0.05). The options including “Type III” and “Wald Chi-square Test” (WCT) are selected for modeling.

## References

[1] Pickett S.T.A., White P.S. (1985). The Ecology of Natural Disturbance and Patch Dynamics.

[2] Kern C.C., D’Amato A.W., Strong T.F. (2013). Diversifying the composition and structure of managed, late-successional forests with harvest gaps: What is the optimal gap size?. For. Ecol. Manag..

[3] Muscolo A., Bagnato S., Sidari M., Mercurio R. (2014). A review of the roles of forest canopy gaps. J. For. Res..

[4] Gray A.N., Spies T.A., Pabst R.J. (2012). Canopy gaps affect long-term patterns of tree growth and mortality in mature and old-growth forests in the Pacific Northwest. For. Ecol. Manag..

[5] Zhang M.M., Wang Z.Y., Liu X.L., Yi X.F. (2017). Seedling predation of *Quercus mongolica* by small rodents in response to forest gaps. New For..

[6] Dai X. (1996). Influence of light conditions in canopy gaps on forest regeneration: A new gap light index and its application in a boreal forest in east-central Sweden. For. Ecol. Manag..

[7] Canham C.D., Denslow J.S., Platt W.J., Runkle J.R., Spies T.A., White P.S. (1990). Light regimes beneath closed canopies and tree-fall gaps in temperate and tropical forests. Can. J. For. Res..

[8] Ni X.Y., Berg B., Yang W.Q., Li H., Liao S., Tan B., Yue K., Xu Z.F., Zhang L., Wu F.Z. (2018). Formation of forest gaps accelerates C, N and P release from foliar litter during 4 years of decomposition in an alpine forest. Biogeochemistry.

[9] González G., Lodge D.J., Richardson B.A., Richardson M.J. (2014). A canopy trimming experiment in Puerto Rico: The response of litter decomposition and nutrient release to canopy opening and debris deposition in a subtropical wet forest. For. Ecol. Manag..

[10] Yan Q., Gang Q., Zhu J. (2019). Size-dependent patterns of seed rain in gaps in temperate secondary forests, Northeast China. Forests.

[11] Sezen U.U., Chazdon R.L., Holsinger K.E. (2005). Genetic consequences of tropical second-growth forest regeneration. Science.

[12] Lai H.R., Hall J.S., Turner B.L., Van Breugel M. (2017). Liana effects on biomass dynamics strengthen during secondary forest succession. Ecology.

[13] Li Y., Jiao J., Wang Z., Cao B., Wei Y., Hu S. (2016). Effects of Revegetation on Soil Organic Carbon Storage and Erosion-Induced Carbon Loss under Extreme Rainstorms in the Hill and Gully Region of the Loess Plateau. Int. J. Environ. Res. Public Health.

[14] Jia G.M., Cao J., Wang C., Wang G. (2005). Microbial biomass and nutrients in soil at the different stages of secondary forest succession in Ziwulin, Northwest China. For. Ecol. Manag..

[15] Scheiner S.M. (1988). Population dynamics of an herbaceous perennial danthonia spicata during secondary forest succession. Am. Midl. Nat..

[16] Chen X.J., Wu X., Yuan Z.Q., Chen X., Zhang Y.W., Cao C.X. (2017). Spectral characteristics and species identification of rhododendrons using a discriminative restricted boltzmann machine. Spectrosc. Lett..

[17] Zhang J., Ma Y., Wu Z., Dong K., Zheng S., Wang Y. (2017). Natural hybridization and introgression among sympatrically distributed rhododendron species in Guizhou, China. Biochem. Syst. Ecol..

[18] Römer A.H., Kneeshaw D.D., Bergeron Y. (2007). Small gap dynamics in the southern boreal forest of Eastern Canada: Do canopy gaps influence stand development?. J. Veg. Sci..

[19] Duguid M.C., Frey B.R., Ellum D.S., Kelty M., Ashton M.S. (2013). The influence of ground disturbance and gap position on understory plant diversity in upland forests of southern New England. For. Ecol. Manag..

[20] Prescott C.E., Hope G.D., Blevins L.L. (2003). Effect of gap size on litter decomposition and soil nitrate concentrations in a high elevation spruce-fir forest. Can. J. For. Res..

[21] Muscolo A., Sidari M., Mercurio R. (2007). Variations in soil chemical properties and microbial biomass in artificial gaps in silver fir stands. Eur. J. For. Res..

[22] Özcan M., Gökbulak F. (2015). Effect of size and surrounding forest vegetation on chemical properties of soil in forest gaps. iForest.

[23] Schliemanna S.A., Bockheim J.G. (2011). Methods for studying treefall gaps: A review. For. Ecol. Manag..

[24] Tsui C.C., Chen Z.S., Hsieh C.F. (2004). Relationships between soil properties and slope position in a lowland rain forest of southern Taiwan. Geoderma.

[25] Zhang Z., Hu B., Hu G. (2014). Spatial heterogeneity of soil chemical properties in a subtropical karst forest, southwest China. Sci. World J..

[26] Sisira E., Bmp S., Marks A. (2008). Variation in canopy structure, light and soil nutrition across elevation of a Sri Lankan tropical rain forest. For. Ecol. Manag..

[27] Oliveira-Filho A.T., Curi N., Vilela E.A., Carvalho D.A. (1998). Effects of canopy gaps, topography, and soils on the distribution of woody species in a central Brazilian deciduous dry forest. Biotropica.

[28] Hejcmanovā-Nežerková P., Hejcman M. (2006). A canonical correspondence analysis (CCA) of the vegetation–environment relationships in Sudanese Savannah, Senegal. S. Afr. J. Bot..

[29] Chou S.C., Huang C.H., Hsu T.W., Wu C.C., Chou C.H. (2010). Allelopathic potential of *Rhododendron formosanum* Hemsl in Taiwan. Allelopath. J..

[30] Nelson D.W., Sommers L.E. (1982). Total Carbon, Organic Carbon and Organic Matter. Methods of Soil Analysis Part 2.

[31] Lin X., Feng Y., Zhang H., Chen R., Wang J., Zhang J., Chu H. (2012). Long-term balanced fertilization decreases arbuscular mycorrhizal fungal diversity in an arable soil in North China revealed by 454 pyrosequencing. Environ. Sci. Technol..

[32] Jiao X., Teng Y., Zhan Y., Wu J., Lin X. (2015). Soil heavy metal pollution and risk assessment in Shenyang Industrial District, Northeast China. PLoS ONE.

[33] Jobidon R., Cyr G., Thiffault N. (2004). Plant species diversity and composition along an experimental gradient of northern hardwood abundance in picea mariana plantations. For. Ecol. Manag..

[34] Clarke K.R., Warwick R.M. (2001). Change in Marine Communities: An Approach to Statistical Analysis and Interpretation.

[35] Archer K.J., Kimes R.V. (2008). Empirical characterization of random forest variable importance measures. Comp. Stat. Data Anal..

[36] Field A. (2005). Discovering Statistics Using SPSS.

[37] Scharenbroch B.C., Bockheim J.G. (2008). Gaps and soil C dynamics in old growth northern hardwood-hemlock forests. Ecosystems.

[38] Arunachalam A., Arunachalam K. (2000). Influence of gap size and soil properties on microbial biomass in a subtropical humid forest of northeast India. Plant Soil.

[39] Zederer D.P., Talkner U., Spohn M., Joergensen R.G. (2017). Microbial biomass phosphorus and C/N/P stoichiometry in forest floor and A horizons as affected by tree species. Soil Biol. Biochem..

[40] Bates T.E., Johnston R.W. (1991). Soil Acidity and Liming.

[41] Scharenbroch B.C., Bockheim J.G. (2007). Impacts of forest gaps on soil properties and processes in old growth northern hardwood-hemlock forests. Plant Soil.

[42] He Z.S., Liu J.F., Su S.J., Zheng S.Q., Xu D.W., Wu Z.Y., Hong W., Wang J.L.M. (2015). Effects of forest gaps on soil properties in *Castanopsis kawakamii* nature forest. PLoS ONE.

[43] Behera S.K., Shukla A.K. (2014). Spatial distribution of surface soil acidity, electrical conductivity, soil organic carbon content and exchangeable potassium, calcium and magnesium in some cropped acid soils of India. Land Degrad. Dev..

[44] Whitehead D.C., Dibb H., Hartley R.D. (1981). Extractant pH and the release of phenolic compounds from soils, plant roots and leaf litter. Soil Biol. Biochem..

[45] Wang C.M., Li T.C., Jhan Y.L., Weng J.H., Chou C.H. (2013). The impact of microbial biotransformation of catechin in enhancing the allelopathic effects of *Rhododendron formosanum*. PLoS ONE.

[46] Inderjit, Mallik A.U. (1997). Effect of phenolic compounds on selected soil properties. For. Ecol. Manag..

[47] Young C.C. (1984). Non-polar macroreticular resin to recover phenolic acids from a subtropical latosol. Soil Biol. Biochem..

[48] Wurzburger N., Hendrick R.L. (2007). Rhododendron thickets alter N cycling and soil extracellular enzyme activities in southern Appalachian hardwood forests. Pedobiologia.

[49] Griffths R.P., Gray A.N., Spies T.A. (2010). Soil properties in old-growth Douglas-fir forest gaps in the western Cascade Mountains of Oregon. Northwest Sci..

[50] He J., Yang W., Xu L., Ni X., Li H., Wu F. (2015). Copper and zinc dynamics in foliar litter during decomposition from gap center to closed canopy in an alpine forest. Scand. J. For. Res..

[51] Muscolo A., Sidari M., Mercurio R. (2007). Influence of gap size on organic matter decomposition, microbial biomass and nutrient cycle in Calabrian pine (Pinus laricio Poiret) stands. For. Ecol. Manag..

[52] Sharma L.N., Grytnes J.A., Måren I.E., Vetaas O.R., Paruelo J. (2016). Do composition and richness of woody plants vary between gaps and closed canopy patches in subtropical forests?. J. Veg. Sci..

[53] Yao A.W., Chiang J.M., McEwan R., Lin T.C. (2015). The effect of typhoon-related defoliation on the ecology of gap dynamics in a subtropical rain forest of Taiwan. J. Veg. Sci..

[54] Terborgh J., Huanca Nuñez N., Alvarez Loayza P., Cornejo Valverde F. (2017). Gaps contribute tree diversity to a tropical floodplain forest. Ecology.

[55] Ranjitkar S., Luedeling E., Shrestha K.K., Guan K., Xu J. (2012). Flowering phenology of tree rhododendron along an elevation gradient in two sites in the Eastern Himalayas. Int. J. Biometeorol..

[56] Taneda H., Kandel D.R., Ishida A., Ikeda H. (2016). Altitudinal changes in leaf hydraulic conductance across five Rhododendron species in Eastern Nepal. Tree Physiol..

[57] Miln R.I., Abbott R.J. (2008). Reproductive isolation among two interfertile Rhododendron species: Low frequency of post-F1 hybrid genotypes in alpine hybrid zones. Mol. Ecol..

[58] Mcclure J.W., Lee T.D. (1993). Small-scale disturbance in a northern hardwoods forest: Effects on tree species abundance and distribution. Can. J. For. Res..

[59] Rao P., Barik S.K., Pandey H.N., Tripathi R.S. (1997). Tree seed germination and seedling establishment in treefall gaps and understorey in a subtropical forest of northeast India. Austral. Ecol..

[60] Burton J.I., Mladenoff D.J., Forrester J.A., Clayton M.K., Gilliam F. (2014). Experimentally linking disturbance, resources and productivity to diversity in forest ground-layer plant communities. J. Ecol..

[61] Tang J., Davy A.J., Jiang D., Musa A., Wu D., Wang Y.C., Miao C.P. (2016). Effects of excluding grazing on the vegetation and soils of degraded sparse-elm grassland in the Horqin sandy land, China. Agr. Ecosyst. Environ..

[62] Crouzeilles R., Ferreira M.S., Chazdon R.L., Lindenmayer D.B., Sansevero J.B.B., Monteiro L., Iribarrem A., Latawiec A.E., Strassburg B.B.N. (2017). Ecological restoration success is higher for natural regeneration than for active restoration in tropical forests. Sci. Adv..

[63] Reid J.L., Fagan M.E., Zahawi R.A. (2018). Positive site selection bias in meta-analyses comparing natural regeneration to active forest restoration. Sci. Adv..

